# Delivery of germacrone (GER) using macrophages-targeted polymeric nanoparticles and its application in rheumatoid arthritis

**DOI:** 10.1080/10717544.2022.2044936

**Published:** 2022-02-28

**Authors:** Tingfei Tan, Qi Huang, Weiwei Chu, Bo Li, Jingjing Wu, Quan Xia, Xi Cao

**Affiliations:** a Department of Pharmacy, The First Affiliated Hospital of Anhui Medical University, Hefei, People’s Republic of China; b The Grade 3 Pharmaceutical Chemistry Laboratory of State Administration of Traditional Chinese Medicine, Hefei, People’s Republic of China; c Department of Oncology, The Second Affiliated Hospital of Anhui Medical University, Hefei, China

**Keywords:** Germacrone, nanoparticles, macrophage-targeted, macrophage polarization, rheumatoid arthritis

## Abstract

Macrophages can transform into M1 (pro-inflammatory) and M2 (anti-inflammatory) phenotypes, which mediate the immune/inflammatory response in rheumatoid arthritis (RA). Activated M1 phenotype macrophages and overexpression of folate (FA) receptors are abundant in inflammatory synovium and joints and promote the progression of RA. Germacrone (GER) can regulate the T helper 1 cell (Th1)/the T helper 2 cell (Th2) balance to delay the progression of arthritis. To deliver GER to inflammatory tissue cells to reverse M1-type proinflammatory cells and reduce inflammation, FA receptor-targeting nanocarriers loaded with GER were developed. In activated macrophages, FA-NPs/DiD showed significantly higher uptake efficiency than NPs/DiD. *In vitro* experiments confirmed that FA-NPs/GER could promote the transformation of M1 macrophages into M2 macrophages. In adjuvant-induced arthritis (AIA) rats, the biodistribution profiles showed selective accumulation at the inflammatory site of FA-NPs/GER, and significantly reduced the swelling and inflammation infiltration of the rat's foot. The levels of pro-inflammatory cytokines (TNF-α, IL-1β) in the rat's inflammatory tissue were significantly lower than other treatment groups, which indicated a significant therapeutic effect in AIA rats. Taken together, macrophage-targeting nanocarriers loaded with GER are a safe and effective method for the treatment of RA.

## 1. Introduction

Rheumatoid arthritis (RA) is an autoimmune disease with a global incidence of similarly 1%. It is characterized by aggressive inflammation of multiple joints of the hands and feet, causing pain and restricting movement (Lu et al., 2018). As the disease worsens, the risk of cartilage destruction and bone damaging increases, thereby resulting in severe disability (Crielaard et al., 2012; Wang et al., 2017). Although the etiology of RA is unclear, studies have shown that macrophages play a crucial role in the pathogenesis of RA (Kinne et al., 2000). Activated macrophages (M1) can secrete large quantity of inflammatory cytokines (tumor necrosis factor-α, TNF-α and interleukin-1β, IL-1β), leading to the progression of RA (Udalova et al., 2016; Xia et al., 2018). In contrast, macrophages (M2) act as anti-inflammatory agents and actively participate in tissue repair. Therefore, converting the M1 macrophages in arthritic joints into M2 macrophages may be an attractive method for the treatment of RA (Jang et al., 2013; Jain et al., 2015).

Currently, the treatment drugs for RA mainly include non-steroidal anti-inflammatory drugs (NSAIDs), glucocorticoids (GCs), etc. (Abbasi et al., 2019). However, the severe adverse effects of these agents have greatly affected people's clinical application, especially for long-term medication (Vezmar et al., 2003; Dixon et al., 2010; Smolen et al., 2010). Recently, the use of plants to prevent and treat arthritis and other chronic diseases has aroused great interest in people. For example, Rhizoma curcuma, a traditional Chinese herbal medicine, is usually adopted to treat inflammation and cancer (Rodrigo et al., 2010). Many essential oils of Rhizoma curcuma are the main biologically active ingredients with anti-inflammatory and anti-tumor properties (Makabe et al., 2006; Li et al., 2009). Germacrone (GER) is a natural bioactive compound extracted from Rhizoma curcuma essential oils (Yang et al., 2005; Li et al., 2009). Studies have shown that GER has anticancer, antioxidant, and antiviral activities (Yang et al., 2006; Liu et al., 2013; Lim et al., 2016). GER can regulate the T helper 1 cell (Th1)/the T helper 2 cell (Th2) balance and inhibit nuclear factor kappa-B (NF-κB) signaling in RA to delay the progression of arthritis (Wu et al., 2016). Moreover, GER reduces the expression of pro-inflammatory cytokines IL-6 and TNF-α, promoting the expression of anti-inflammatory mediators transforming growth factor-β (TGF-β) and interleukin-10 (IL-10), and protecting neonatal rats from acute lung injury caused by lipopolysaccharide (LPS) (Wang et al., 2019). However, plant-derived medicine faces challenges in drug delivery and target effects, which may be due to its poor stability and solubility, and rapid elimination from the body systems (An et al., 2014; Mehta et al., 2016; Singh et al., 2019). There is an urgent need to develop an effective delivery system to continuously deliver the active ingredients to the desired site of action while keeping the toxicity to a minimum (Rajeshkumar et al., 2019; Wadhwa et al., 2019).

Nanotechnology has proven to be an ideal choice by replacing traditional therapies and overcoming problems related to drug delivery and toxicity (Bosch, 2011; Nikalje, 2015). Recently, nanoparticle (NP)-based drug delivery systems (DDSs) have been developed for targeted therapy of RA (Hofkens et al., 2011; Sahoo et al., 2017). Compared with traditional drugs, designing NPs as drug carriers offer abundant advantages including drug delivery improvement at the target site or targeted cell, low toxicity, relatively low dose, and protecting the drug from degradation and digestion before reaching the active site *in vivo* (Gouveia et al., 2015; Jahangirian et al., 2017). In addition, NPs also have unique physical properties, which can be transferred to inflammation areas and tumor sites through the loose vascular system to achieve therapeutic effects (Jin et al., 2018). For example, using polycaprolactone (PCL)–polyethylene glycol (PEG) micelles to encapsulate low-dose GCs could reduce inflammation, the anti-inflammatory effect was better than that of free dexamethasone and had mild side effects (Wang et al., 2016). Crielaard et al. showed that encapsulating GCs into core cross-linked polymer micelles enhanced the treatment of RA by improving drug release (Crielaard et al., 2012). Over the years, according to specific needs, nanotechnology has been transformed from original nanomaterials into various modifications (Hu et al., 2017, 2018). Zhao et al. developed folate modified NPs containing methotrexate and folate receptors β (FRβ). Activated macrophage binding significantly inhibited the occurrence and development of RA in adjuvant-induced arthritis (AIA) rats (Zhao et al., 2017). Sun et al. showed that NPs with FR targeting ligand on the surface had high affinity for FRβ on macrophages, resulting in significant therapeutic effect of NPs on AIA rats (Sun et al., 2019).

In this study, we developed FA modified NPs loaded with GER. The nanocarriers were composed of poly(lactic-co-glycolic acid) (PLGA)–PEG–folic acid (FA), sodium deoxycholate (SDC), and solutol HS15 (HS15). The *in vivo* efficacy was then evaluated in a rat model of AIA.

## 2. Materials and methods

### 2.1. Antibodies and biochemical reagents

Germacrone (purity ≥ 99%), Chengdu Herbipurify Co., LTD (Chengdu, China); SDC and solutol HS15, Sigma-Aldrich (St. Louis, MO); PLGA-PEG-FA, Xian Ruixi Biological Technology Co. Ltd. (Xian, China); incomplete Freund’s adjuvant (10 mg/mL), Chondrex (Washington, DC). 1,1′-Dioctadecyl-3,3,3′,3′-tetramethylindocarbocyanine (DiD),4′,6-diamidino-2-phenylindole (DAPI), and LPS were supplied by Solarbio Science & Technology Co., Ltd. (Beijing, China). CD163 antibody was purchased from BioLegend (San Diego, CA). Antibodies against CD206 and IFN-γ were obtained by Proteintech (Wuhan, PR China). Anti-IL-1β antibody and TNF-α antibody were purchased from Abcam (Cambridge, UK). ELISA kits were obtained from Elkbiotech (Wuhan, China).

### 2.2. Cell culture

RAW 264.7 (mouse Abelson murine leukemia virus-induced tumor) cells were purchased from Chinese Academy of Sciences (Shanghai, China) and maintained in RPMI 1640 medium (HyClone, Logan, UT) supplemented with 10% fetal bovine serum (HyClone, Logan, UT), 1% (v/v) penicillin/streptomycin (Solarbio Science & Technology, Beijing, China) and incubated at 37 °C with 5% CO_2_.

### 2.3. Experimental animals

Male Sprague-Dawley (SD) rats (200 ± 20 g) were provided by the Experimental Animal Center of Anhui Medical University (Hefei, China). The rats were housed in common animal rooms and fed standard feed, with an indoor temperature of 25 ± 2 °C and a relative humidity of 50 ± 10%. All experimental protocols described in this study were approved by the Ethics Committee of Anhui Medical University.

### 2.4. Preparation of drug-loaded nanoparticles

GER (5 mg) and PLGA-PEG (10 mg) were dissolved in chloroform and placed in a round bottom flask. In a 37 °C water bath, the organic solvent was removed by a rotary evaporator to form a uniform lipid film. SDC and HS15 were dissolved in 5 mL of distilled water and added, and the flask was placed at 37 °C in an air bath shaking table. The film was removed by hydration, and then the probe was ultrasonicated at 250 W power for 3 min (every 3 s, 5 s rest). The resulting general NPs were loaded with GER. FA-NPs/GER were composed of GER (5 mg), PLGA-PEG (10 mg), FA-PEG-PLGA (10 mg) SDC and HS15, and the other operating steps were the same as described above.

### 2.5. Characteristics of drug-loaded nanoparticles

At room temperature, we used the Malvern-Zeta sizer Nano S90 (Malvern Instruments, Malvern, UK) to measure the size and zeta potential of the NPs. The morphology of NPs was analyzed by transmission electron microscopy (H600, Hitachi, Shiga, Japan). In terms of stability, our NPs were added to PBS (ZSGB-BIO, Beijing, China) and serum (Biological Industries, Beit Haemek, Israel), and the stability was observed at 4 °C and 37 °C. The changes of particle size were recorded at 0, 2, 4, 8, 10, and 24 hours. The encapsulation efficiency (EE) and drug loading efficiency (DLE) were determined by HPLC.

### 2.6. Drug release from GER nanoparticles *in vitro*

The release of GER in NP_S_ and FA-NPs was studied by dialysis. Four milliliters of NPs were transferred to the dialysis bags (molecular weight cutoff, 10 kDa) and the dialysis bags was placed in 40 mL of phosphate-buffered saline (PBS) containing 0.2% (w/v) tween 80. Free GER solution was used as the control. One milliliter of release medium was taken at the specified time and immediately replenished the same volume of fresh release medium to ensure that the total volume of release medium remained constant. The concentration of the released drug was detected by HPLC (Waters, Milford, MA).

### 2.7. Cellular uptake studies

RAW 264.7 cells in logarithmic growth phase were seeded at 1 × 10^5^ cells/well and inoculated in 12-well plates. Activated macrophages (M1-type) were generated with LPS (1 µg/mL) and IFN-γ (20 ng/mL), and nonactivated macrophage, cultured at 37 °C for 24 hours. The medium was discarded, and the cells were washed twice with sterile PBS. DiD solution, NPs/DiD, and FA-NPs/DiD (an equivalent dose of 5 μg/mL DiD) were added to the wells and incubated with 264.7 cells for 0.5 and 2 hours. The cells suspension was collected, centrifuged at 3000 rpm for 3 min, and the supernatant was discarded. After the cells were washed with PBS for three times, they were resuspended in PBS, and the fluorescence intensity was measured by flow cytometry (Beckman Coulter, Brea, CA). Subsequently, the cells were fixed in 4% paraformaldehyde (20 min), permeabilized in 0.5% Triton X-100 (15 min), DAPI staining (5 min), and observation under a confocal laser scanning microscope (CLSM) (Leica, Wetzlar, Germany).

### 2.8. Establishment of adjuvant-induced arthritis rat model

SD rats were fed normally for one week before administration, and 0.1 mL 10 mg/mL complete Freund's adjuvant was injected subcutaneously into their tail root. The establishment of arthritis was observed every day. The thickness of swollen claws was measured 2 weeks after adjuvant injection. Rats with obvious inflammation were selected as AIA rats.

### 2.9. Biodistribution and macrophage targeting of nanoparticles in arthritic rats

DiD is a lipophilic fluorescent molecule with good stability *in vivo*. Arthritic rats were injected intravenously with DiD solution, NPs/DiD and FA-NPs/DiD (an equivalent dose of 70 μg/kg DiD). At 2 h, 8 h, and 24 h after the tail vein injection, the rats were anesthetized by 10% chloral hydrate (0.3 mL/100 g) and the dynamic fluorescence distribution was observed using the intravital imaging System FX PRO (excitation wavelength 644 nm, emission wavelength 665 nm, X-ray exposure time 20 s) (Bruker, Billerica, MA).

### 2.10. Therapeutic efficacy of nanoparticles in arthritic rats

On days 16, 18, 20, 22, and 24 after arthritis induction, normal saline, GER, NPs/GER, and FA-NPs/GER were injected (an equivalent dose of 8 mg/kg GER) into the tail vein of each group (*n* = 6). Six healthy rats that did not receive any treatment served as blank controls. Starting from the 16th day after arthritis induction, the soles of all animals were evaluated for thickness and swelling every other day. The rats were sacrificed on the 26th day after the induction of arthritis, and blood and joint tissues were collected. The pro-inflammatory cytokines (TNF-α, IL-1β) in serum of each group (3000 rpm centrifugation for 5 min) were detected by ELISA kit. Western blot and PCR detected the levels of pro-inflammatory cytokines (TNF-α, IL-1β) in the joint tissues of each group.

### 2.11. Regulation of macrophage polarization *in vitro*

RAW264.7 cells were transformed into M2-type macrophages (M2) under stimulation with interleukin-4 (IL-4), which served as a positive control. We divided the cells into five groups: PBS group, GER group, NPs/GER group, FA-NPs/GER group, and M1-type macrophages (LPS– and IFN-γ-stimulated RAW 264.7 cells) incubated for 3 h. M1 and M2 macrophages were labeled with CD86 antibody and CD206 antibody, respectively. The ability of M1macrophages to transform into M2 macrophages was investigated by laser confocal microscopy.

### 2.12. Biosafety assessment of nanoparticles

With healthy rats as the control group, the body weight of AIA rats was measured on the 16th, 18th, 20th, 22nd, 24th, and 26th days after induction. Then, the rats were killed and the important organs such as heart, liver, spleen, lung, and kidney were taken. It was fixed in 4% paraformaldehyde for 24 hours and embedded in paraffin for pathological section. Sections were stained with hematoxylin and eosin (H&E) to evaluate the safety of the preparation

### 2.13. Statistical analysis

Statistical analysis was performed using SPSS 26.0 (IBM, Chicago, IL). All results were expressed as mean ± SD. Student’s *t*-test was used to compare the differences between means of two groups, and one-way ANOVA was used to compare the means of three or more groups to determine whether they differ significantly from one another. *p*<.05 was considered statistically significant.

## 3. Results

### 3.1. Characterization and stability of drug-loaded nanoparticles

**Figure 1.**
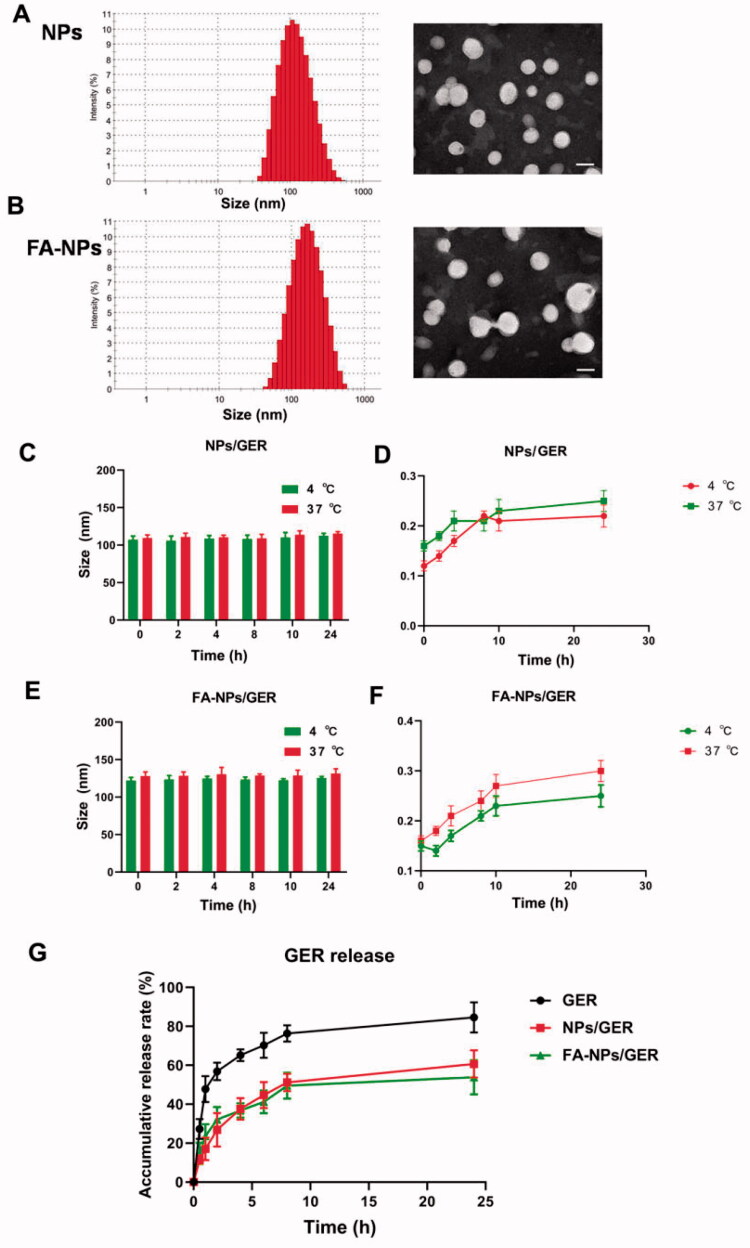
Physicochemical characterization of nanoparticles. (A) Size distributions and TEM images of NPs. (B) Size distributions and TEM images of FA-NPs. (C) *In vitro* stability of NPs/GER at 4 °C or 37 °C. (D) Serum stability of NPs/GER at 4 °C or 37 °C. (E) *In vitro* stability of FA-NPs/GER at 4 °C or 37 °C. (F) Serum stability of FA-NPs/GER at 4 °C or 37 °C. (G) *In vitro* release profiles of GER, NPs/GER, and FA-NPs/GER at 37 °C in PBS. Data represent mean ± SD (*n* = 3).

The mean diameters of the NPs and FA-NPs were less than 200 nm, as measured by dynamic light scanning (Table 1). Figure 1(A,B) shows that the particle sizes of FA-NPs were close to those of NPs, and FA modification had little effect on the particle size of NPs. Transmission electron microscopy showed that all types of NPs were uniformly dispersed and spherical, with a particle size of 100–200 nm.

**Table 1.** Characterization of NPs/GER and FA-NPs/GER.

Nanoparticles	Size (nm)	Zeta potential (mV)	EE (%)	DL (%)
NPs/GER	138.7 ± 5.2	–24.9 ± 3.9	87.3 ± 2.2	2.3 ± 0.3
FA-NPs/GER	144.3 ± 6.6	–31.2 ± 3.4	85.5 ± 1.3	2.4 ± 0.3

Data represent mean ± SD (*n* = 3).

By measuring the particle size of the NPs at different temperatures in PBS and in serum, we could preliminarily determine the stability of the NPs. These data, as shown in Figure 1(C–F), were continuously monitored for 24 h, regardless of the location at 37 °C or 4 °C. The particle size and PDI of NPs/GER and FA-NPs/GER did not change significantly. There was no obvious aggregation or fusion after 24 h, which indicated that the NPs had good stability.

### 3.2. Drug release from nanoparticles *in vitro*

Compared with NPs, free drug showed a faster release rate, and the cumulative release *in vitro* reached 80% in the first 8 h. In the NPs/GER group and FA-NPs/GER group, the cumulative release of GER was only approximately 50%, even after 24 h. FA-NPs/GER could not release drugs rapidly and prevented drug leakage to a certain extent (Figure 1(G)).

### 3.3. Cellular uptake efficiency

**Figure 2.**
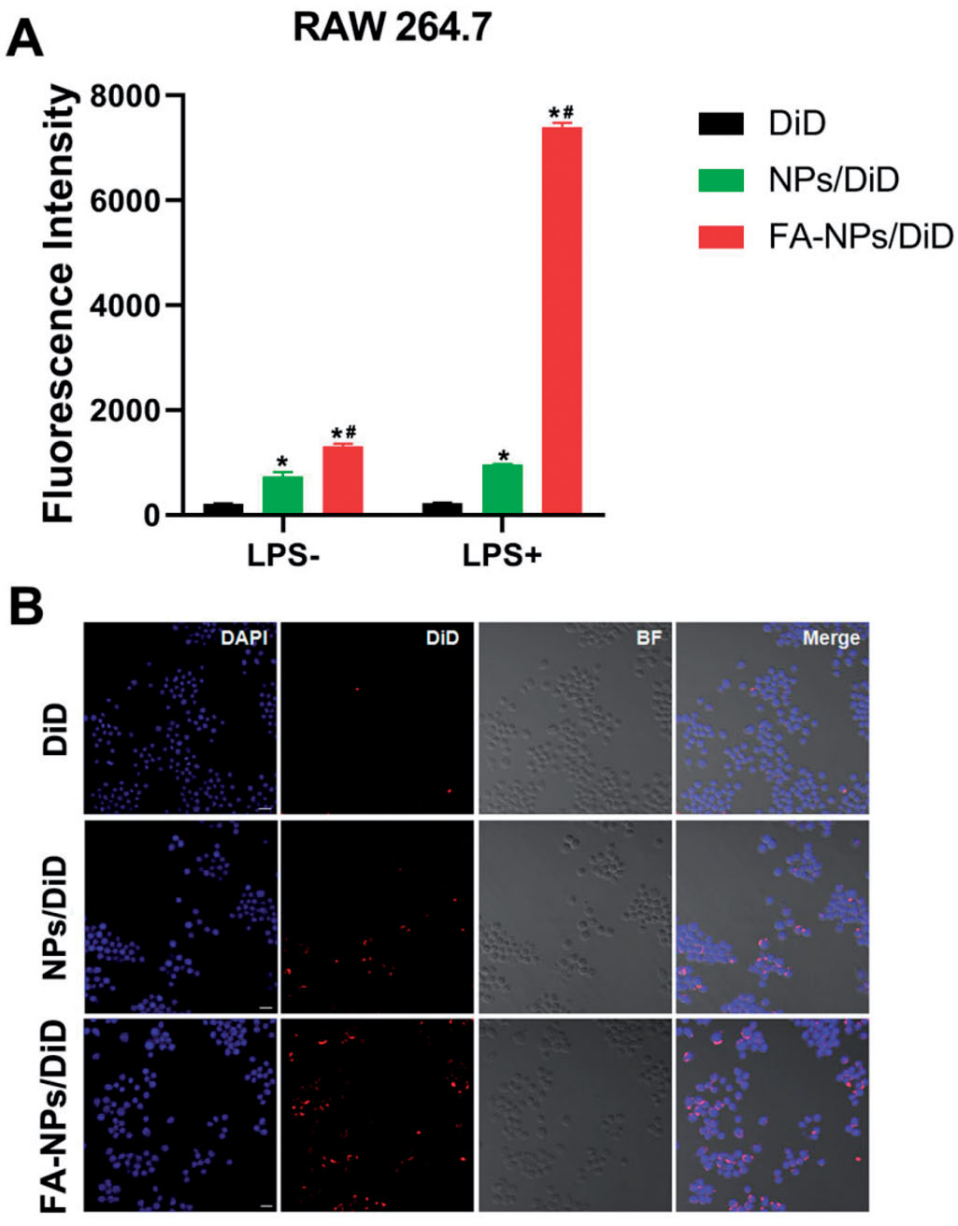
Cellular uptake behaviors. (A) Flow cytometry images of cellular uptake in RAW 264.7 (LPS–) and RAW 264.7 (LPS+) at 2 h with the listed treatments. (B) Confocal laser scanning microscopy images of M1 macrophages. Cell nuclei were stained with DAPI (blue), and DiD fluorescence is displayed in red. Scale bar represents 100 μm. *p<.05 vs. DiD. ^#^*p*<.05 vs. NPs/DiD. Data represent mean ± SD (*n* = 3).

In order to evaluate whether FA-NPs could be specifically taken up by inflammatory cells, the DiD was used instead of GER. NPs/DiD and FA-NPs/DiD were prepared as described in the ‘Materials and methods’ section. In our experiments, we selected activated and un-activated RAW 264.7 cells. According to the results of flow cytometry, in RAW 264.7 cells, both NPs and FA-NPs showed an increase in fluorescence intensity over time (Figure 2(A,B)). The uptake efficiency of FA-NPs/DiD in 264.7 cells was significantly higher than that of NPs/DiD (*p*<.05). In addition, the uptake efficiency of FA-NPs/DiD (LPS+) was significantly higher than that of FA-NPs/DiD (LPS–). Consistent with the CLSM results, the intracellular DiD fluorescence distribution in the FA-NPs group was significantly enhanced compared with the NPs/DiD group.

### 3.4. Biodistribution and macrophage targeting of nanoparticles in arthritic rats

**Figure 3.**
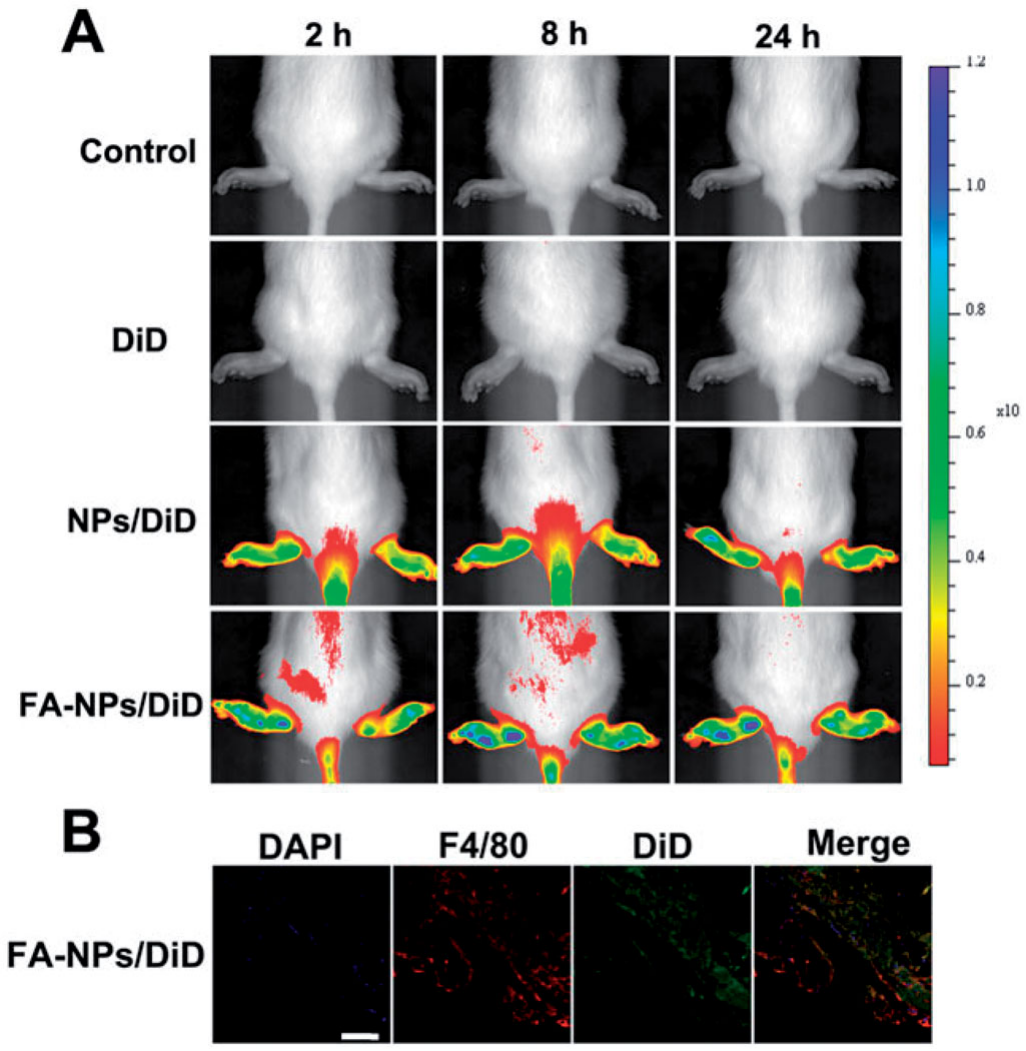
*In vivo* biodistribution of NPs/DiD in rats with adjuvant-induced arthritis. (A) Real-time fluorescence images of control, free DiD, NPs/DiD, and FA-NPs/DiD at different time points. (B) Confocal laser scanning microscopy images of legs treated with FA-NPs/DiD for 24 h. Cell nuclei were stained with DAPI (blue), F4/80 fluorescence is displayed in red, and DiD fluorescence is displayed in green. Scale bar represents 100 μm.

To observe the distribution of NPs, DiD was loaded onto NPs. The results showed increased fluorescence intensity in rat paws in the NPs and FA-NPs groups compared with free DiD (Figure 3(A)). The FA-NPs group exhibited stronger and longer fluorescence intensity in rat paws than the NPs group. The CLSM images showed that the NPs not only gathered in the joints but also targeted the macrophages in the joints (Figure 3(B)). Biodistribution studies had shown that FA-NPs targeted the inflamed soles of rats and prolong the action time of the drug.

### 3.5. Therapeutic efficacy of nanoparticles *in vivo*

**Figure 4.**
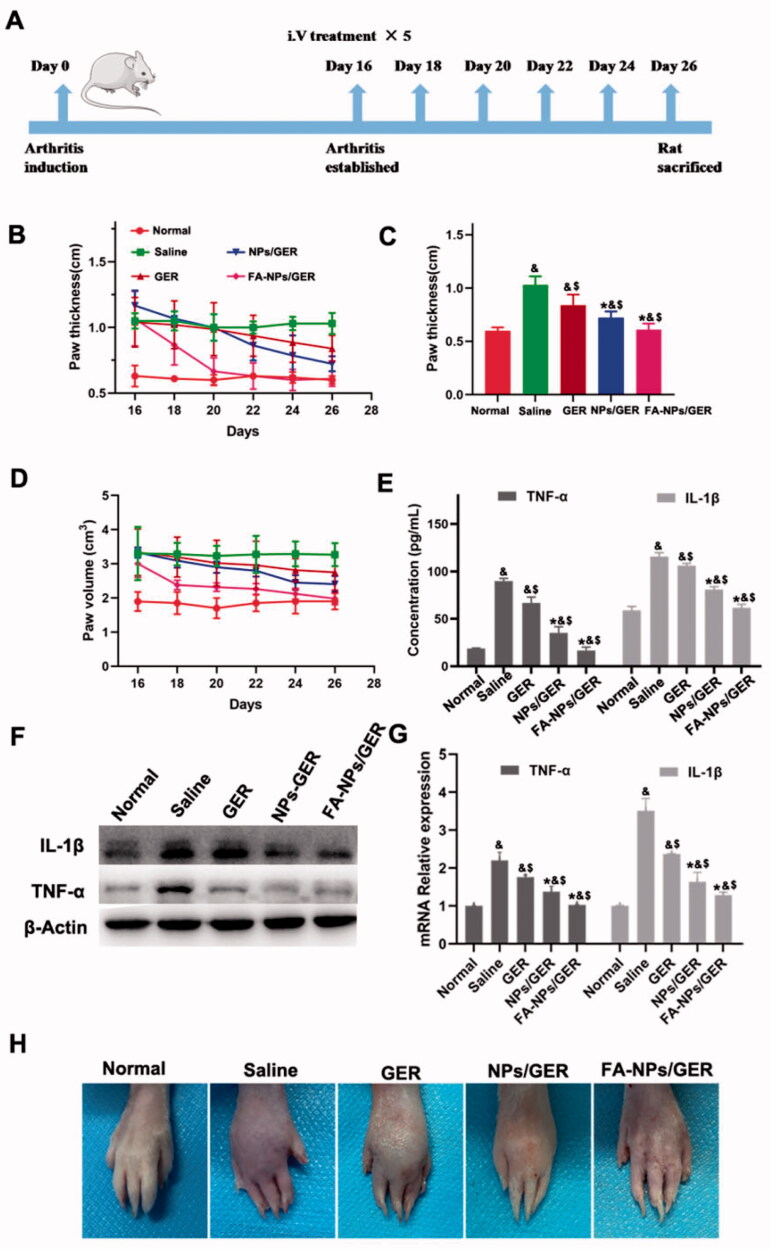
Arthritis induction in rats and assessment of therapeutic effects with the listed treatments. (A) Schematic diagram of arthritis model establishment and intravenous administration. (B) Hind paw thickness variation trend. (C) Hind paw thickness. (D) Hind paw volume. (E) Concentration of TNF-α and IL-1β in serum. (F) GER treatment downregulated the expression of TNF-α and IL-1β (*n* = 3). (G) The relative expression levels of TNF-α and IL-1β in the hind paw. (H) Photographs of representative hind legs from different treatment groups. ^&^*p*<.05 vs. normal. ^$^*p*<.05 vs. saline. *p<.05 vs. GER. The results are presented as the mean ± SD (*n* = 6).

On the 16th day after induction, the AIA rat model was successfully constructed (Figure 4(A)). The anti-inflammatory effects of free GER, NPs/GER, and FA-NPs/GER on AIA rats were studied by photographing the hind paws, measuring the thickness of hind paws and measuring the volume of hind paws. In Figure 4(B–D), remarkable differences in paw thickness and paw volume were found between the FA-NPs/GER-treated group and the other two groups (*p<.05 vs. GER ^#^*p*<.05 vs. NPs/GER. The cytokines TNF-α and IL-1β drove the progression of RA. The levels of both proinflammatory cytokines in serum were significantly lower in animals treated with FA-NPs/GER than in animals in the other groups (*p*<.05) (Figure 4(E)). These results were verified by WB and PCR experiments. The levels of FA-NPs/GER inflammatory factors were significantly lower than those in the other groups (Figure 4(F,G)). These results suggested greater therapeutic efficacy with FA-NPs/GER than with NPs/GER and free GER. Figure 4(H) shows hind paw images of all groups. Compared with the normal group, the foot swelling and erythema were observed in the free GER solution group. For the FA-NPs/GER treatment group, there was no obvious swelling or erythema in the paws. These results indicated that FA-NPs/GER is an effective formulation for the treatment of RA.

### 3.6. Regulation of macrophage polarization *in vitro*

**Figure 5.**
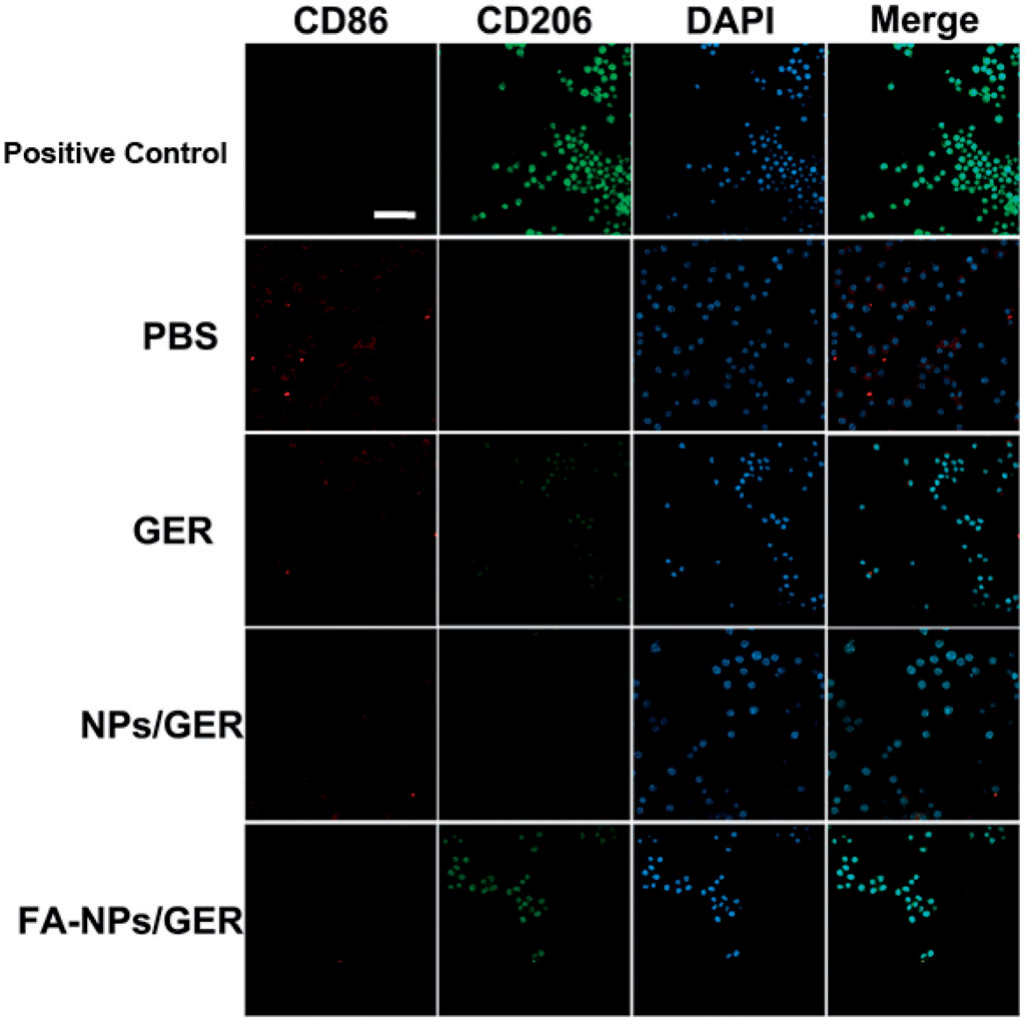
Confocal laser scanning microscopy images of macrophages after treatment with PBS, GER, NPs/GER, and FA-NPs/GER. Cell nuclei were stained with DAPI (blue), CD86 fluorescence displayed in red and CD206 fluorescence displayed in green. Scale bar represents 20 μm. Data represent mean ± SD (*n* = 3).

Confocal laser scanning microscopy showed that the number of M1 macrophages in PBS group was the highest (Figure 5). In the given group, the number of M1 macrophages gradually decreased and the number of M2 macrophages gradually increased in GER, NPs/GER and FA-NPs/GER groups. M2 macrophages in the FA-NPs/GER group were similar to those in the positive group. *In vitro* results confirmed that FA-NPs/GER could promote the transformation of M1 macrophages into M2 macrophages.

### 3.7. Biosafety assessment of nanoparticles

**Figure 6.**
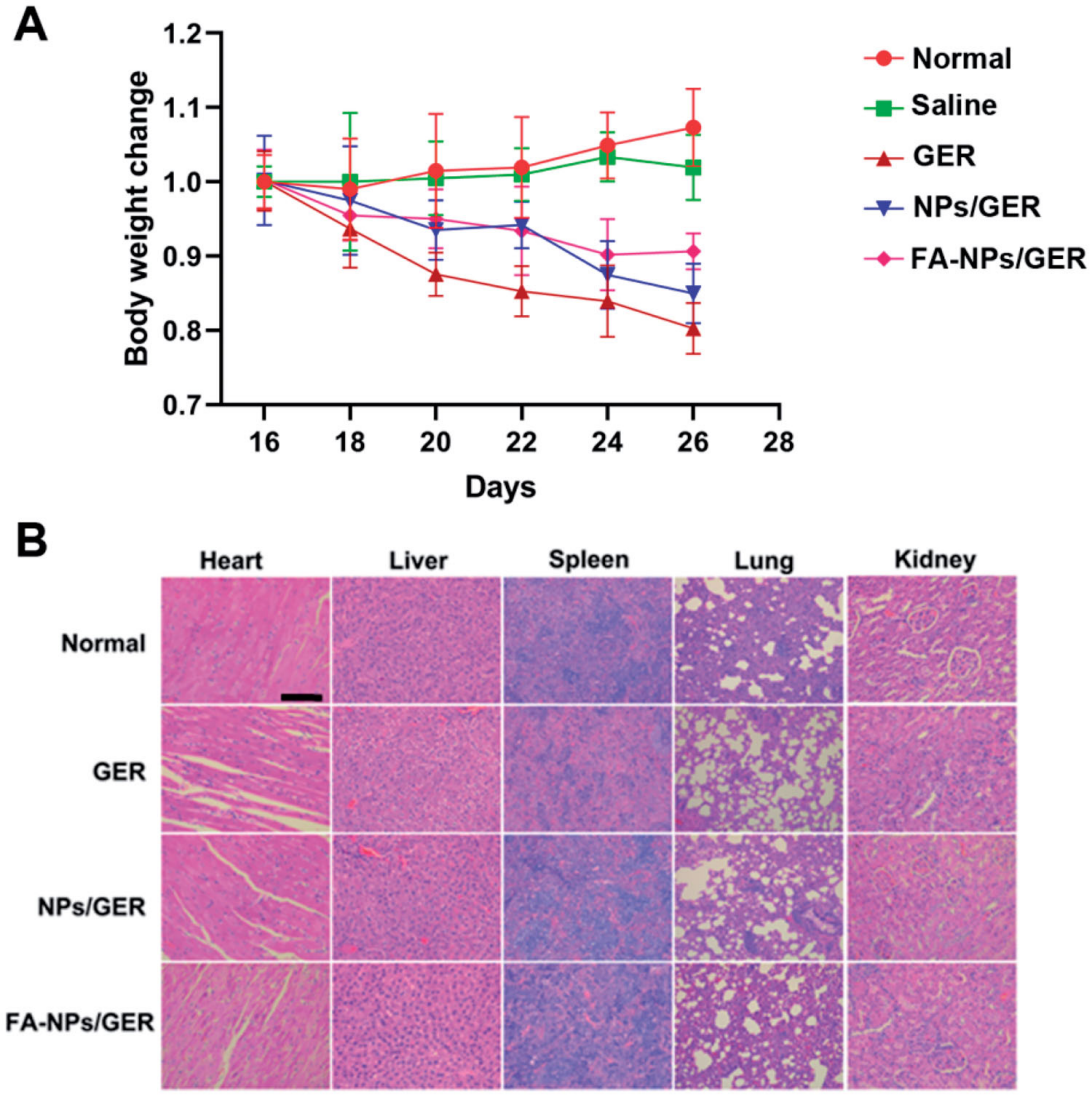
*In vivo* toxicity of nanoparticles in AIA rats. (A) Body weight variations after treatment over time. (B) H&E staining and histological analysis images were obtained in major organs and tissues. Those are representative sections from five rats analyzed for each group. Scale bar = 100 μm.

According to the body weight records, we observed that the weight of all the administration groups decreased, and the weight of the GER group decreased significantly. However, the weight change of FA-NPs/GER was the smallest (Figure 6(A)). The sectioned organs of rats in the four groups also comparatively investigated the systematic toxicity by H&E staining. Compared with the control group, there were no significant pathological changes in various organs (heart, liver, spleen, lung, and kidney) in FA-NPs/GER group (Figure 6(B)). These results showed that FA-NPs/GER had good safety in the treatment of RA.

## 4. Discussion

At present, the main purpose of treatment for RA is to achieve the highest anti-inflammatory effect while minimizing joint damage. Germacrone is one of the major bioactive components separated from Rhizoma curcuma (Aggarwal et al., 2013). Various studies have shown the anti-inflammatory roles of GER. Makabe et al. (2006) showed that GER could inhibit three quarters of ear inflammation. An et al. (2014) showed that GER could significantly suppress pro-inflammatory cytokines and increased the expression of anti-inflammatory factors. Through GER treatment, LPS-promoted tissue damaged in newborn rats was also reduced, further indicating that GER had an anti-inflammatory effect.

Due to the variety in their physicochemical properties, NPs have been investigated as carriers and DDSs. Advances in nanotechnology, chemistry, and biology have accelerated the emergence of nanomedicines (NMs) (Ferrari et al., 2015; Liang et al., 2018; Zhang et al., 2018). Recently, enhanced permeability and retention (EPR) effect have been exploited to passively target NMs to RA joints (Yang et al., 2017; Zhang et al., 2018). Growing evidence has suggested that an EPR-like phenomenon is also a sign of RA, because with the progress of inflammation, the blood–joint barrier is destroyed, leading to vascular leakage, and the endothelial cell space in the inflammation joints is as high as 600 nm (Ochoa & Stevens, 2012; McInnes & Schett, 2017). Compared with passive targeting, active targeting enhances NP-uptake and optimizes sustained drug-delivery (Dolati et al., 2016).

Macrophage infiltration is an obvious characteristic of inflammatory disease. Macrophages activity levels are closely associated with the development of RA, the activation of M1 macrophages to produce a variety of inflammatory cytokine proteins to maintain and increase the formation of joint inflammation and activate M2 macrophages secreted anti-inflammatory cytokines to combat this condition. Studies have demonstrated that folate receptors (FRs) are overexpressed in activated macrophages, and the specific affinity between FRs and FA provides an opportunity to achieve active target drug delivery by FA-modified NPs (Nogueira et al., 2016; Chen et al., 2017; Chandrupatla et al., 2019). Bilthariya et al. showed that active targeting offers appropriate advantages over passive targeting by enhancing NP absorption and optimizing sustained drug delivery. Therefore, this strategy can be used as an effective therapeutic alternative to reduce drug dose and increase biocompatibility (Bilthariya et al., 2015). In our research, we developed FA-modified NPs-loaded GER (FA-NPs/GER). The FA-NPs/GER had a size of 144.3 ± 6.6 and a smooth spherical structure under TEM. The drug releasing *in vitro* illustrated that FA-NPs/GER could not release drugs rapidly, which was suitable for accumulation in inflammatory tissues.

In RA, the formation of synovial hyperplasia and vasospasm may cause early cartilage and bone erosion. Studies have found that their interface contains a large number of activated macrophages, which was known as M1 macrophages. These macrophages continue to secrete a variety of pro-inflammatory cytokines (such as TNF-α and IL-1β) to drive the progression of RA. This makes macrophages a potential therapeutic target for inflammatory diseases (Kinne et al., 2000; Lawrence & Natoli, 2011). In our study, LPS-activated macrophages were used as an inflammation model to explore the cellular-uptake efficiency of NPs. At all given time points, the uptake efficiency of FA-NPs/DiD in RAW 264.7 cells were significantly higher than that of NPs/DiD and the uptake efficiency of FA-NPs/DiD (LPS+) were significantly higher than that of FA-NPs/DiD (LPS–). These results showed that FA-modified NPs might be an effective carrier. Biodistribution studies had shown that FA-NPs/GER targeted the inflamed ankle joint and prolonged the action time of GER. In addition, *in vitro* experiments also confirmed that FA-NPs/GER reversed the conversion of activated macrophages (M1) to M2 macrophages and reduced the expression of pro-inflammatory factors. Finally, we constructed an AIA rat model to investigate the therapeutic effects of FA-NPs/GER. The results showed that FA-NP/GER treatment led to a significant improvement in the clinical outcomes of paws. The lower levels of proinflammatory cytokines (TNF-α and IL-1β) confirmed the inflammatory inhibition of FA-NPs/GER. Compared with the normal group, the foot swelling and erythema were observed in the free GER solution group. For the FA-NPs/GER treatment group, there was no obvious swelling or erythema in the paws. In our study, the body weight of rats and HE staining of various organs showed the safety of FA-NPs/GER. This provides new ideas for the use of GER in the treatment of RA. Overall, the results suggested that FA-NPs/GER have a significant therapeutic effect on AIA rats and could treat RA by regulating the transformation of M1 macrophages to M2 macrophages.

In summary, our study reported FA-modified NPs loaded with GER (FA-NPs/GER) for the first time and successfully used them for the treatment of AIA rats. FA-NPs/GER could release the drug slowly, thus increasing the delivery of GER to the target inflammatory tissue. Our experiments showed better accumulation in inflammatory tissues via the FR-targeting of FA-NPs/GER and demonstrated promotion of the transformation of M1 macrophages into M2 macrophages to reduce inflammation. Thus, FA-NPs/GER may be a safe drug to slow down the progression of inflammation. The discovery of FR expression in macrophages raises the possibility of FA-NPs/GER as a target clinical treatment of RA (Yya et al., 2020).

## Data Availability

The authors confirm that the data supporting the findings of this study are available within the article.
